# The Place of the Bifactor Model in Confirmatory Factor Analysis Investigations Into Construct Dimensionality in Language Testing

**DOI:** 10.3389/fpsyg.2020.01357

**Published:** 2020-07-17

**Authors:** Karen J. Dunn, Gareth McCray

**Affiliations:** ^1^Assessment Research Group, British Council, London, United Kingdom; ^2^School of Primary, Community and Social Care, Keele University, Keele, United Kingdom

**Keywords:** language testing, psychometrics, bifactor model, higher-order model, dimensionality, confirmatory factor analysis (CFA)

## Abstract

For practical and theoretical purposes, tests of second language (L2) ability commonly aim to measure one overarching trait, general language ability, while simultaneously measuring multiple sub-traits (e.g., reading, grammar, etc.). This tension between measuring uni- and multi-dimensional constructs concurrently can generate vociferous debate about the precise nature of the construct(s) being measured. In L2 testing, this tension is often addressed through the use of a higher-order factor model wherein multidimensional traits representing subskills load on a general ability latent trait. However, an alternative modeling framework that is currently uncommon in language testing, but gaining traction in other disciplines, is the bifactor model. The bifactor model hypothesizes a general factor, onto which all items load, and a series of orthogonal (uncorrelated) skill-specific grouping factors. The model is particularly valuable for evaluating the empirical plausibility of subscales and the practical impact of dimensionality assumptions on test scores. This paper compares a range of CFA model structures with the bifactor model in terms of theoretical implications and practical considerations, framed for the language testing audience. The models are illustrated using primary data from the British Council’s Aptis English test. The paper is intended to spearhead the uptake of the bifactor model within the cadre of measurement models used in L2 language testing.

## Introduction

Dimensionality considerations are important for both the development and ongoing validation of tests of second language (L2) ability. For practical and theoretical purposes, language tests are commonly designed to measure one overarching trait, that of general L2 ability, while simultaneously measuring multiple sub-traits (usually L2 reading, listening, speaking, writing). Items are written with the aim of assessing these highly related but conceptually distinct abilities. It is crucial for a strong validity argument that test constructors are able to isolate and examine the similarities and differences between various L2 skill areas. Indeed, the meaningful evidence-based delineation and reporting of scales and possible subscales and their appropriate usage is an essential aspect in making a construct validity argument for a test (Slocum-Gori and Zumbo, 2010). This has particular ramifications for practical decisions regarding score reporting. Where guidelines are given regarding sub-scores, the requirement for sufficiently high reliability and distinctiveness for all scores is emphasized (e.g., AERA et al., 2014). When reporting a test score on a single scale, the implication is that the test is measuring one unitary skill or trait, and that the scores given reflect the candidate’s ability or level on that single trait. Splitting the test into sub-scores and reporting these separately indicates each sub-score should require a sufficiently distinct aspect of ability from the other sub-scales. From a theoretical perspective, an understanding of language tests as straddling both uni- and multi-dimensional structures is now a generally accepted viewpoint within the academic language testing community. Harsch’s (2014) “state of play” summary on dimensionality in L2 language testing emphasizes that “language proficiency can be conceptualized as both unitary *and* divisible, depending on the level of abstraction and the purpose of the assessment and score reporting” (Harsch, 2014, p. 153). Nonetheless, achieving a balance between these concurrent theorizations can generate sometimes vociferous debate about the precise nature of, and relationship between, the construct(s) measured.

This paper will compare the kinds of insights various confirmatory factor analysis (CFA) models can offer into the underlying dimensional structure of sets of test items designed to tap into different but highly related knowledge domains. Four model structures are described and discussed: the unidimensional model, the correlated traits model, the higher- (or second-) order model, and the bifactor model. The first three are frequently used in analysis of L2 language tests, while the fourth, the bifactor model, is less commonly employed in this field for analysis. The ultimate aim of the paper is to gauge what added value the bifactor model can bring to the assessment of dimensionality and, thus, to place its usefulness in the language test researcher’s CFA toolkit. Two illustrative studies are presented, which employ language testing datasets, plus a brief literature review on the background to the dimensionality debates surrounding each area addressed. The first illustrative study examines the evidence for the divisibility and, thus, the appropriateness of sub-score reporting, of a grammar and vocabulary test component with 50 items, 25 intended to measure grammar and 25 intended to measure vocabulary. The second illustrative study examines the evidence for multidimensionality in data representing the traditional four skills (L2 listening, reading, speaking, and writing) comprising an overall measure of general second language (L2) ability. The abovementioned psychometric models will be fitted to the data and then interpreted. It is important to note that while this paper does fit a battery of models, which address common debates in L2, the focus here is primarily on demonstrating the modeling and inferential process, particularly regarding the bifactor model, rather than generalizing theory from the substantive interpretations of the results. Furthermore, note that this paper will illustrate why using only an assessment of model fit statistics to choose the most appropriate form for score reporting is a limited and inappropriate analytical strategy. The theoretical, statistical, and practical differences between the four models will be discussed, and recommendations for usage in the language testing context will be provided.

## Literature Review

### Dimensionality of Large-Scale Language Tests

Over the past decade and beyond, work exploring the dimensionality of a range of large-scale language tests has supported the interpretation of multi-skill tests as comprising a series of strongly related, yet distinct, dimensions. A large body of such studies have employed CFA techniques to show either the correlated factor or higher-order factor model to be the most appropriate model to represent the underlying measurement qualities of the test in question (Shin, 2005; Stricker et al., 2005; Sawaki et al., 2009; In’nami and Koizumi, 2012; Sawaki and Sinharay, 2013, 2017; In’nami et al., 2016; Kim and Crossley, 2019). Usually, a CFA study involves proposing various theoretically informed structures for the relationships between sets of items purported to be measuring different dimensions. Statistical models are then fitted to collected data, which operationalize these theoretical structures, and evidence is gathered on which of the models best describes the data and, thus, which of the structures is most likely closest to that under which the data was generated. The tests in these analyses included: TOEIC,^®^ which was found to be best represented by a correlated factor model for reading and listening (In’nami and Koizumi, 2012); the TEAP test, represented by a higher-order model for the four skills (In’nami et al., 2016); the TOEFL iBT^®^ meanwhile has been subject to a large number of studies with a higher-order model being favored in some projects (Stricker and Rock, 2008; Sawaki et al., 2009) and the correlated four-factor model in others (Sawaki and Sinharay, 2013, 2017).

Several of the studies explored the use of a bifactor model as a possible representation of a multidimensional structure hypothesized to underlie a test (Sawaki et al., 2009; Sawaki and Sinharay, 2017). This modeling framework is currently uncommon in language testing, but gaining traction in other disciplines (Reise, 2012). The bifactor model incorporates a general factor, onto which all items load directly, plus a series of orthogonal (i.e., specified as uncorrelated) factors each loading on a sub-set of items (Reise, 2012). Where the higher-order and correlated factor models account for commonalities within and across each of the subscales, the bifactor model explicitly models the general commonality between all items in the test and the residual variance for each skill area beyond that of general L2 proficiency, with equal weight (see below for further details). It is important to note, however, that statistically, the higher-order model has been shown to be nested within the bifactor model (Yung et al., 1999; Rijmen, 2010; Markon, 2019). The subordinate factors in a higher-order model mediate the relationship with the more general factor, but the higher-order factor can be expressed in terms of their direct relationship with the observed variables following mathematical transformation. The two models are not, therefore, as far removed from one another as it may first appear, however, employing the more flexible bifactor model has implications for interpretation of multidimensionality in language tests as shall be explored in this paper.

The aim of the majority of the language testing studies cited above exploring the dimensionality of large-scale language tests is to justify score reporting practices, which break down an overall score into a number of sub-scores for each skill area. As observed by Sawaki and Sinharay (2017), “conceptual distinctness among section scores does not necessarily guarantee their psychometric distinctness from one another” (Sawaki and Sinharay, 2017, p. 530–531). The importance of these studies is to provide empirical backing for theoretical assumptions about the underlying structure of L2 language tests. This is of particular interest to test developers, since stakeholders often expect detailed feedback and are perhaps not overly concerned with its justification from a measurement perspective. It is, therefore, unsurprising to find that few of the studies explored alternative groupings of the sub-scales in the tests. In other words, because of the influence of stakeholders, scores are often reported in a traditional way, e.g., an overall score with reading, listening, reading, and writing sub-scores, whether or not the subscales are shown to be psychometrically distinct. There are, however, a couple of exceptions to this rule. One example is Kim and Crossley’s (2019) investigation of the latent structure of the Examination for the Certificate of Competency in English (ECCE) across test sections, addressing ability in reading, listening, writing, speaking, and lexico-grammatical ability (Kim and Crossley, 2019). These researchers identified a three-factor solution, with one factor representing reading, listening, and lexico-grammar, and the additional two factors representing writing and speaking abilities, respectively. In addition, they found this structure to hold across age and gender sub-groups of the data. Another example of an alternative factor structure was presented by Stricker et al. (2005), whose modeling of the LanguEdge test showed speaking to load on one factor while reading, listening, and writing all jointly loaded on a second. From a measurement perspective, when an alternative structure is indicated for a test, there will be an implication for score reporting. However, stakeholder expectations may be resistant to, or ultimately prohibit, changes in this regard owing to the use of language sub-scores in decision-making processes. This point is emphasized by Sawaki and Sinharay (2017), who focused in their study on the degree to which section scores can offer value-added information to stakeholders. In addition to investigating the overall factor structure of the TOEFL iBT^®^, these researchers explored the extent to which section sub-scores are reliable and, importantly, distinct, from other sub-scores. These researchers employed a classical test theory-based sub-score analysis (Haberman, 2008). The current paper, meanwhile, discusses how the bifactor model provides a tool to explore such considerations within the CFA framework.

### Dimensionality of Grammar and Vocabulary Tests

Considerations regarding test dimensionality are also pertinent in addressing the distinction between grammar and vocabulary knowledge. While superficially these two aspects of language may seem different, separating the constructs is not as clear-cut is it may first appear. From an analytic perspective, and with much dependent on how the constructs are operationalized, the likelihood is that candidates scoring highly on vocabulary items would have a strong tendency to score highly on grammar items. However, this does not necessarily mean that they are indivisible constructs or that it is desirable to treat them on a unidimensional scale in all cases; indeed, researchers have been mixed in their recommendations on conceptualizing grammar and vocabulary on a uni- or bi-dimensional scale. Taking examples from studies that aim to describe the components of reading ability, it can be seen that Purpura (1999), for instance, drew on both vocabulary and grammar measures to form a single “lexico-grammatical ability” factor, while Shiotsu and Weir (2007) and Shiotsu (2010) maintained a distinction.

Test developers need to be sensitive to the manner in which the grammar and vocabulary constructs are operationalized in any given test before assuming a united or divided treatment of these language knowledge areas. This notion was demonstrated by Alderson and Kremmel (2013), who warned about the need to be cognizant of the “slipperiness of the slope” between grammar and vocabulary knowledge, and for the test constructor be able to define and defend their decisions to report the constructs separately (Alderson and Kremmel, 2013, p. 550). In addition, it should be recognized that grammar and vocabulary may well be activated differently within each language domain. For example, while readers can rely on linguistic information in the text via bottom–up processes, the “online” nature of listening means that learners tend to draw more on top–down processes (Lund, 1991; Park, 2004). In practical terms, this means that the listener will perhaps compensate for lack of specific vocabulary knowledge by drawing on other, more general or metacognitive, areas of knowledge, but this is less common in reading (van Zeeland and Schmitt, 2013, p. 461). Consequentially, when considering the theoretical arguments for the dimensionality of grammar and vocabulary, one should carefully consider the specific operationalization of the constructs and not make overgeneralizations that grammar and vocabulary are always *or* never distinct.

Data from measures underpinned by such closely related constructs, and which tap into such tightly interrelated knowledge domains, very often result in item responses that are consistent with both unidimensional and multidimensional interpretations (Reise et al., 2010). Described as a “dimensionality quagmire” by researchers working in clinical psychology settings (Reise et al., 2018), a similar state of affairs is equally applicable to the language testing context. The choice of measurement model is, nonetheless, crucial for both score reporting and assessing score reliability (Brunner et al., 2012). It then becomes the job of the researcher to take into account information from a range of sources when considering the dimensionality of a test, of which statistical evaluation is just one aspect.

### Current Aims and Research Question

The aim of the current paper is to illustrate, in some detail, the usefulness of a range of factor analytic models in answering questions about test dimensionality. While most of the models will be familiar to L2 language test researchers working with CFA, the intention here is to encourage the integration of the bifactor model into the cadre of models already employed by academics and test developers in this field. Two key points are emphasized in this paper:

1. CFA models are to be viewed as tools used to gather evidence, rather than truth-makers. *Inferences on dimensionality should not be solely based on statistical fit any more than they should be purely based on expert judgment of item content.*
2. All tests, and indeed subscales of tests, with more than one distinct item are multidimensional to some extent. *It is the job of the researcher and the test constructor to investigate the tenability of assuming unidimensionality and reporting a single score for these scales and subscales.*

To elaborate on these points, the current paper will describe the way in which information from various models offered within the CFA framework can be used to complement theoretical understandings and practical requirements. The comparative nature of this paper aims to provide a framework for researchers to evaluate what the bifactor model might bring to their assessment of L2 language test dimensionality in addition to the oft-used models. Particular focus is given therefore to interpretation and applicability of the bifactor model which, as a less commonly used model, is more vulnerable to misinterpretation, particularly of the meaning of the trait-specific factors (DeMars, 2013). The following research question is addressed via two worked examples, using data from two variants of the British Council’s Aptis test:

RQ: What insights useful to both test and theory developers can the: (i) unidimensional; (ii) correlated factors; (iii) higher-order; and (iv) bifactor model provide about the dimensionality and score interpretation of the underlying construct(s) when applied to L2 language test data?

## Model Descriptions

Each of the models employed in the worked examples below are introduced in the following four sub-sections.

### Unidimensional Model

**FIGURE 1:**
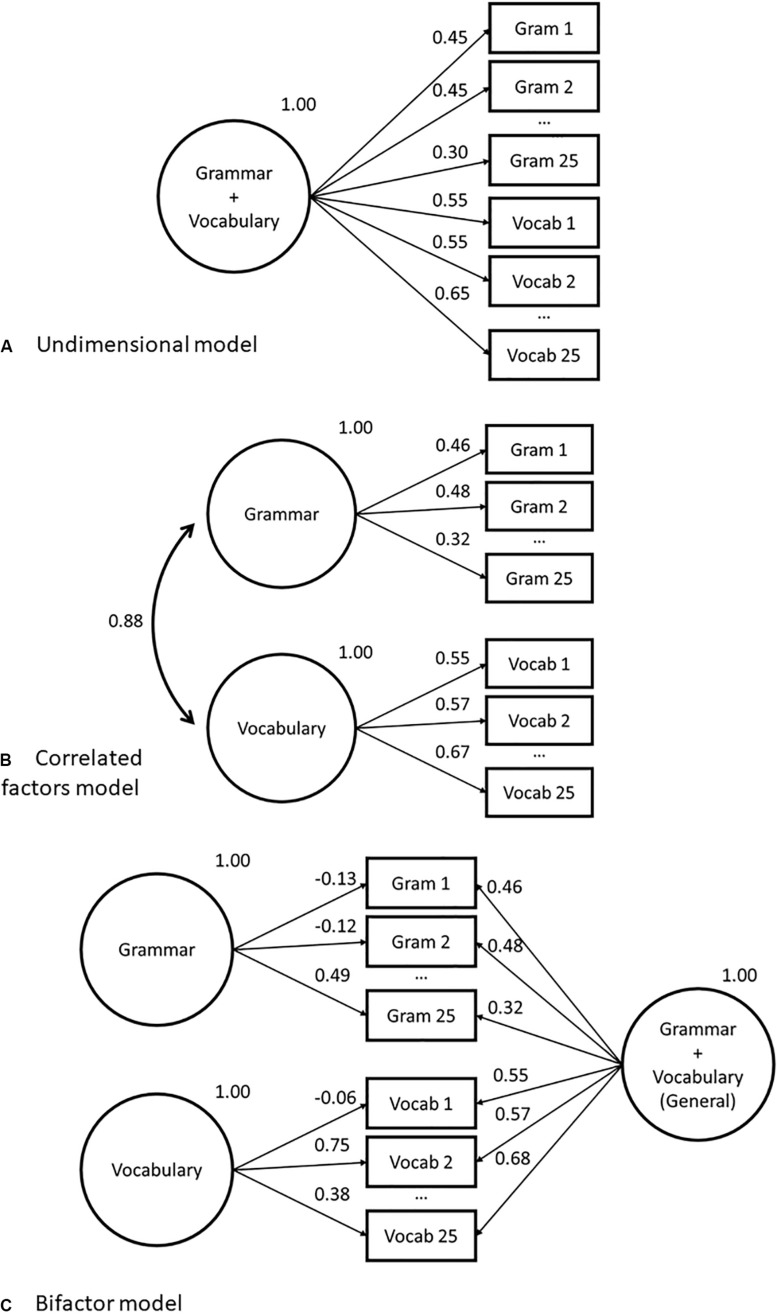
Abbreviated factor loading diagrams for confirmatory factor analysis (CFA) models fit to grammar and vocabulary dataset in study 1. Models as follows: **(A)** Unidimensional; **(B)** Correlated factors; **(C)** Bifactor.

**FIGURE 2:**
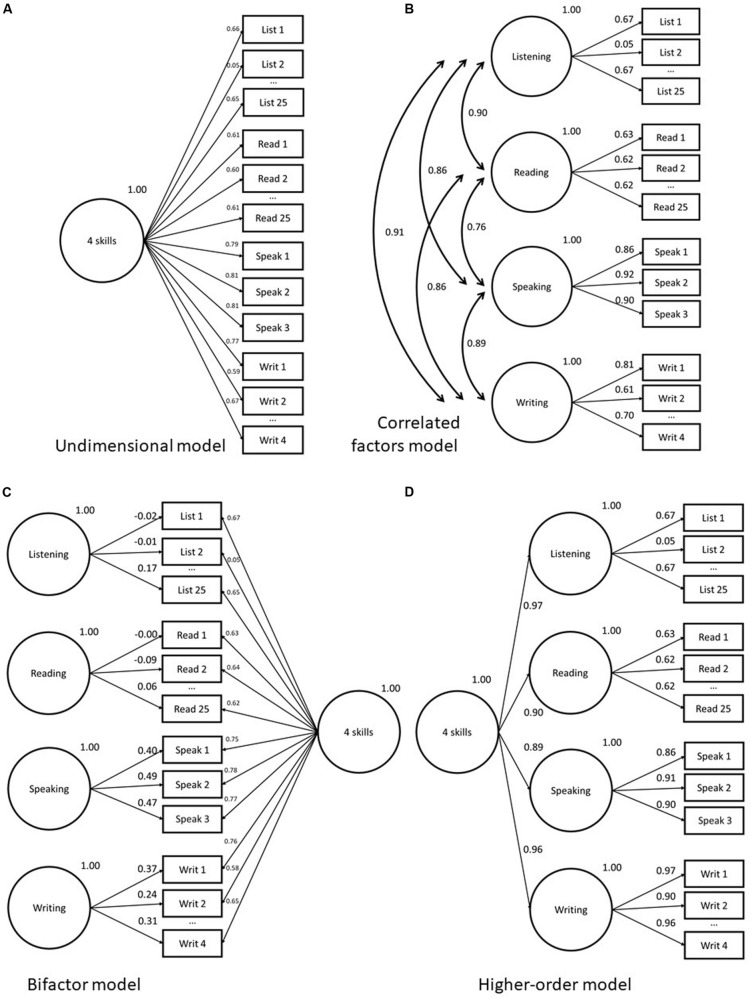
Abbreviated factor loading diagrams for CFA models fit to four-skill dataset in study 2. Models as follows: **(A)** Unidimensional; **(B)** Correlated factors; **(C)** Bifactor; **(D)** Higher-order.

Unidimensionality is a key assumption within almost all scoring models in both classical as well as item response test theory (Gustafsson and Åberg-Bengtsson, 2010). The unidimensional model hypothesizes a single factor to explain the variance across all observed variables (i.e., the variance in test scores across all items), with no differentiation between sub-groups of items. This model is illustrated in Figures 1A, 2A below. A series of estimated loadings indicate the strength of the relationship between the single factor and each of the observed variables. An error term (omitted in the figures in this paper) is also estimated against each observed variable, since the latent factor is not assumed to provide a perfect explanation of the observed variance. Standardized loadings can be directly compared, and smaller loadings on the general factor will be associated with a higher degree of error and, thus, the response to an item providing less information about a test-taker’s trait score.

The unidimensional model is the most commonly applied (or assumed) model in psychometrics, and it is particularly valuable as it can be used to model items measuring various aspects of a construct on the same scale and report a single score to represent the ability of the test taker. A key question to answer when using this model is: “Is this test unidimensional?” Or, in other words, can a large proportion of variance in observed test scores be explained with reference to the same underlying construct? When modeling language test data, this factor is often hypothesized as general L2 ability.

### Correlated Factors Model

The correlated factors model (e.g., Brown, 2015) includes two or more latent variables, which are allowed to correlate (see Figures 1B, 2B for illustrations). Observed variables are grouped by shared features and act as indicators for a factor hypothesized to reflect this commonality. This explicitly models the multidimensionality of a test. The correlated factors model does not incorporate any general or underlying factor, however, the correlations between each of the latent variables indicate shared variation across all pairs of latent variables in the model. A series of loadings indicate the strength of the relationship between the observed variables and their associated factor. Again, error terms are estimated against each observed variable. Note that each observed variable in the model is assumed to be only associated with a single factor.

The correlated factors model is often used as a point of comparison with the unidimensional model described above. A language testing researcher might want to ask: “Is this test multidimensional?” or perhaps he or she will have more specific questions regarding whether a particular group of items constitutes a subscale.

### Higher-Order Model

The higher-order model (Thurstone, 1944) incorporates at least one superordinate (higher-order) factor and a series of subordinate factors upon which specified sub-group of items load (see Figure 2D for an illustration). This second- or higher-order factor explicitly models the shared variance between subordinate factors, meaning that these first-order grouping factors are conditionally independent of one another, and each one mediates the relationship between the overarching, or superordinate, factor and the observed variables.

The higher-order model estimates two sets of loadings: those showing the relationships between the observed variables and the relevant grouping, or subordinate, factor, plus those showing the relationship between the higher-order factor and each of the subordinate factors. Error terms against each of the observed variables show that the model is not hypothesized to perfectly explain the variance of the observed variables, and error terms on the factors (termed *disturbances* in CFA literature) indicate that this higher-order factor does not explain all the variance of each of the subordinate factors.

Higher-order models are often used for theory testing (Brown, 2015), and they enable the researcher to explore theoretical understandings of the relationship between a series of sub-tests as distinct from one another, but also united by a common factor, which attempts to explain the scores in the higher-order factor. The researcher might ask: “Can I justify reporting this multi-skill test as an overall scale?” This is a highly relevant question in language testing, where the researcher or test developer may seek empirical justification for the reporting of an overall score in addition to sub-scores for each language domain incorporated in the test. If the loadings between the higher-order and subordinate factors are satisfactorily high, it can be concluded that there is enough commonality between the sub-skills to justify this reporting both sub-scores and an overall score.

It is important to note that in this model, there is no direct relationship hypothesized between the more general (higher-order) factor and the observed variables. The observed variables act as indicators of the subordinate factors, and therefore, the commonality modeled by the higher-order factor is between the scales already established for each sub-group. This mediating role for the subordinate factors means that the higher-order factor, therefore, represents a “distilled” estimate of general ability rather than a more direct estimate, which accounts for commonalities between all observed variables as per the unidimensional model. This distance is termed by Markon (2019) and others as a “level of abstraction,” with the higher-order model the choice of the researcher for whom the subordinate factors are “theoretically salient” (Markon, 2019, p. 53). In practice, this means that there may be commonalities between items across different subscales that are not captured by the higher-order model. If, say, individual items across reading, listening, and writing factors depended, in part, on a particular aspect of knowledge (for example, the “past-perfect tense”), the higher-order model may not see those items load as high on the general factor, after distillation, as they would on a unidimensional model, or indeed the bifactor model, described below.

### Bifactor Model

The bifactor model (Holzinger and Swineford, 1937), also described as a nested-factor (NF) model (Gustafsson and Åberg-Bengtsson, 2010; Brunner et al., 2012), or a hierarchical model (Markon, 2019), incorporates a general factor, which loads directly onto all of the observed variables in the model and, in addition to this, grouping factors, which load onto sub-groups of the same set of observed variables (see Figures 1C, 2C for an illustration. One of the defining features of the bifactor model is that the grouping factors in the model are hypothesized to be orthogonal (uncorrelated) with the general factor. Grouping factors, themselves, can be either correlated or uncorrelated (Reise et al., 2018); however, the focus in this paper is on bifactor models with uncorrelated grouping factors, as providing a more readily interpretable solution. Additionally, unlike the CFA model structures presented above, the bifactor model does not offer a “simple structure” solution in which each observed variable only loads onto a single factor (Gustafsson and Åberg-Bengtsson, 2010). Observed variables, by design, in this model load onto more than one factor, meaning that the variance explanation is split between (at least) two latencies. Each observed variable in the bifactor model is an indicator of both the general factor and one grouping factor. This means that each observed variable has two loading estimates in the model; the first will show its relationship with the general factor and the second with its allocated grouping factor.

While the interpretation of the loadings on the general factor can be understood as per the single factor in the unidimensional model, it is important to note that the estimates for the grouping factors in the bifactor model are *not* analogous to the subordinate factor loadings in the higher-order model or the skill-specific factors in the correlated factors model. The grouping factors in the bifactor model give an estimate of the shared variance between sub-groups of items once the common variance between all observed variables captured by the general factor has been partitioned out. This can be thought of as the relationship between residuals^1^. With respect to scoring considerations, DeMars (2013) described how constructing a group factor score for the bifactor model can be achieved by algebraically combining the loading on the grouping factor and the general factor. Statistical packages do not commonly provide this score by default, as sub-score generation is virtually never the reason a bifactor model is fit. In language testing, a bifactor model of a four-skill test would include the general factor as representative of overall L2 ability, and the grouping factors as representative of a shared aspect of knowledge within each skill area that is not captured by the information about overall L2 ability. This point is discussed in more detail in the second worked example below.

A key distinguishing feature between the bifactor model and the higher-order model is that the general factor is hypothesized to load directly on each of the observed variables. This grants the general factor greater theoretical salience than the grouping factors, the reverse scenario to that of the higher-order model, which foregrounds the skill-specific factors (Markon, 2019). With respect to the accepted understanding of general L2 language proficiency as both “unitary and divisible” (Harsch, 2014), this distinction in emphasis between the two models are not necessarily at odds with one another. As noted earlier, the higher-order model is nested within the bifactor model. This is illustrated by studies that show the possibilities for expressing the direct relationship between the superordinate factor in a higher-order model and the observed variables via a process known as the Schmid–Leiman transformation (Schmid and Leiman, 1957; Yung et al., 1999). The resulting estimates are structurally equivalent to those of a bifactor model subject to certain constraints (Brunner et al., 2012; Markon, 2019). These two models should perhaps not, therefore, be viewed as competing structures, but rather different means of accounting for the multidimensionality in language tests, the estimates from which are useful in different ways, as explored below.

An important question that the bifactor model can help the researcher to answer is: “Is this test unidimensional enough to be reported on a single scale, and relatedly, does it make sense to also report domain sub-scores?” In some respects, the bifactor model fleshes out the insight gained from the unidimensional model in cases where the researcher knows that there are likely to be dependencies between sub-groups of items within the test. Researchers in other disciplines suggest that this factor structure can, in fact, lead to greater conceptual clarity than alternative CFA model structures (e.g., Chen et al., 2012) and are particularly valuable for evaluating the plausibility of subscales (Reise et al., 2010, 2018).

In summary, each of the four models described above acknowledge some interrelatedness between all items. This is an important requirement when modeling tests that assesses L2 knowledge. Echoing the comparative description of these four-factor analytic models from Brunner et al. (2012), the unidimensional model focuses exclusively on general language ability, and the correlated factors model on specific abilities, while the higher-order and the bifactor models both “consider the ability hierarchy in its entirely, containing a mix of general and specific constructs” (Brunner et al., 2012, p. 813). However, in terms of model estimates (without transformations), the language test researcher will note that there is a different division in terms of the manner the overall commonality between items is addressed. The unidimensional and bifactor models directly model shared variance between observed responses, while the correlated factors and higher-order model mediate this relationship by the inclusion of grouping factors at the individual sub-skills or first-order level. These varying structures accord the researcher different insights into the measurement properties of a test. This is demonstrated in the following sections using two examples of the application of CFA models to the kind of data typically analyzed in language testing. An interpretation of the findings is given, which considers the utility of each model fitted to address subtly different sets of questions about the underlying factor structures of the data and the practical ramifications for test constructors of the inferences drawn from the models.

## Illustrative Study 1 – Aptis General Grammar and Vocabulary

The first illustrative study examines the insights that can be gathered from fitting three models to explain the score variance seen in a selection of grammar and vocabulary items: (i) unidimensional; (ii) correlated factors; and (iii) bifactor model. Note that the higher-order model was not fitted in this study as a model with only two first-order factors (i.e., grammar and vocabulary) loading onto the higher-order factor is not statistically identifiable (Brown, 2015). The models fitted are illustrated in Figure 1.

### Study 1 Dataset

The Grammar and Vocabulary component of the Aptis General test variant (O’Sullivan and Dunlea, 2015) was delivered to a large global population (*N* = 17,227) between April 2018 and June 2019. Representation in the dataset was from more than 60 different countries. Ability levels ranged from pre-A1 to above B2 on the Common European Framework of Reference for Languages (CEFR) (Council of Europe, 2001) according to their Aptis score designation. Each test taker completed 25 grammar items and 25 vocabulary items, scored dichotomously. All items were multiple choice, but the vocabulary items had pooled response options (10 options per each block of five items). A description of the test, including sample questions, are available in the Aptis Candidate Guide (British Council, 2019a).

### Study 1 Method

The three CFA models, unidimensional, correlated factors, and bifactor, were fit to these data using the latent variable modeling software Mplus (Muthén and Muthén, 2017) using robust maximum likelihood (MLR) estimation, analogous to marginal maximum likelihood (MML) estimation in an Item Response Theory framework. Response outcomes were modeled at the individual item level; for each candidate (random missingness aside), this was a series of 50 responses, 25 from each test section.

In terms of evaluating each model, for completeness, the model chi-square values and associated *p*-values are reported; however, note that these should not be relied upon for model acceptance or rejection as they are acutely sensitive to sample size (Schermelleh-Engel et al., 2003; Vandenberg, 2006), and the models described have been fit to a large sample. With respect to global fit, i.e., how well the data fit the predictions of the model, root mean squared error of approximation (RMSEA), and comparative fit index (CFI) are reported. As recommended by Hu and Bentler (1999) we report both an absolute index of fit, the RMSEA, where a model is compared against a perfectly fitting model, and a relative index of fit, the CFI, where a model is compared against a baseline, or null, model. For acceptable fit, the RMSEA should be below 0.06, while the CFI should be greater than 0.95 (Hu and Bentler, 1999). Given that the performance of fit indices can vary according to aspects of the model, it is generally recommended to take into account a range of fit statistics when considering model appropriateness (Brown, 2015). Note, as MLR estimation method does not furnish RMSEA and CFI values, when modeling categorical variables, we report statistics that come from fitting the same model on the same data using the weighted least squares mean and variance adjusted (WSLMV) estimation method in Mplus with both models having probit link functions.

Given that the models are not all nested, some comparative fit measures, i.e., measures that allow us to compare the ability of non-nested models to explain the score variance seen, were also extracted. Specifically, AIC, BIC, and sample-size-adjusted BIC are reported. As a reflection of the practical differences between models, two sets of metrics have been generated: mean absolute error (MAE) of the factor loadings for various latent traits against the unidimensional model. The MAE is a measure of the size of an average difference between a parameter on the unidimensional model and a corresponding parameter from another model fitted to the same data^2^. A low MAE indicates a high similarity between sets of parameter values, and a high MAE indicates the converse. To aid in interpretability, factor scores have been rescaled from having a mean of 0 and an SD of 1, to having a mean of 50 and an SD of 10.

It should be noted that there are also various well-documented statistical methods that can be employed to evaluate the usefulness of several of the models described, for example, the commonly calculated “omega” and the “omega hierarchical.” These statistics provide a model-based approach to assess scale and subscale reliability and are relevant to the bifactor model, and the higher-order model following transformations (Gustafsson and Åberg-Bengtsson, 2010; Brunner et al., 2012; Reise et al., 2013; Rodriguez et al., 2016a,b; Reise et al., 2018). There are also statistics that directly address the question of sub-score utility (Rodriguez et al., 2016a,b). A full review of these methods is beyond the scope of the current paper, but the reader is encouraged to explore the literature.

### Study 1 Results

The fit measures and average loadings for the three models are shown in Table 1. None of the models have non-statistically significant chi-square *p*-values, but this is unsurprising given the large sample size. All models have acceptable levels of fit on the absolute index, RMSEA, but none of the models have acceptable levels of fit on the relative index of fit, the CFI, though at 0.896, the bifactor model is very close to the suggested threshold of 0.9 for a reasonable model. All three indices of comparative fit indicate that the bifactor model is the best fit by some margin, followed by the correlated factors model, then, the unidimensional model.

**TABLE 1:** Grammar and vocabulary fit measures and average loadings.

	**Model descriptive statistics**			
**Fit measure**	**Unidimensional**	**Correlated factors**		**Bifactor**
Chisq	70,533 (1,175), *p* < 0.001	66,999 (1,174), *p* < 0.001		42,169 (1.125), *p* < 0.001
RMSEA	0.059	0.057		0.046
CFI	0.824	0.833		0.896
AIC	868,283	865,840		848,767
BIC	869,058	866,623		850,034
ADJ BIC	868,740	866,302		849,558
**Mean (SD) of factor loadings**				
General	0.54(0.17)	*NA*^a^		0.50(0.19)
Grammar	*NA*^a^	0.47(0.13)		0.21(0.25)
Vocab	*NA*^a^	0.64(0.16)		0.25(0.27)
**Factor correlations**				
Gra-Voc	*NA*^b^	0.88		NA^b^
**Mean absolute error (MAE)^c^ of parameters from those on the unidimensional model**				
		**Gra**	**Voc**	**General**

Factor loadings		0.03	0.01	0.08
Factor scores^d^		0.90	1.18	4.11

**^*a*^Not provided as the factor was not measured by the model. ^*b*^Not provided as no correlation between factors was measured in the model. ^*c*^$M\,A\,E=\frac{\sum \left|x_{i}-y_{i}\right|}{n}$, where x is a parameter for the unidimensional model, y is a corresponding parameter from the model in question, and n is the sum of comparisons. ^*d*^Scores set to mean = 50, SD = 10.**

With respect to loading estimates, Table 1 shows that for the unidimensional model, the average loading on the single general factor is 0.54. For the correlated factors model, the average loading for grammar is 0.47, the average loading for vocab is 0.64, and the correlation between the two traits is 0.88. For the bifactor model, the mean loading on the general factor is 0.50, and then the mean loadings for the grouping factors are much lower at 0.21 and 0.25 for grammar and vocab, respectively. In terms of loadings, there is an MAE of 0.03 and 0.01 difference between the grammar and vocabulary loadings on the unidimensional versus the correlated factors model, respectively. There is a 0.08 MAE between the unidimensional model loading and the general factor of the bifactor model. In terms of scores, when put onto a mean = 50 and *SD* = 10 scale, there is a 0.90 and 1.18 average difference in the scores that would be given by the unidimensional model compared to the correlated factors model, for grammar and vocabulary, respectively. There is an average difference of 4.11 between the scaled scores from the unidimensional model and those from the general factor of the bifactor model. Given the fact that the scale is set to have an SD of 10, these differences are minimal and likely to be no greater than error.

Figures 1A–C give traditional factor loading diagrams for the three models used in this section. Note that they are abbreviated, in that all not all observed variables are displayed. Supplementary Table A1 in the appendix provides the full list of item loadings. Conditional formatting has been applied to the table to help with interpretation, where lighter cells are lower and darker cells are higher values. We can see there is little difference between the unidimensional and correlated factors model loadings. There are a series of items from Vocab 6 to Vocab 15 that have higher loadings (> 0.7) than the rest of the items on the scale. We do see some differences, however, between the aforementioned two models and the bifactor model loadings. In the bifactor model, we do not see an overall uniform loading on the grouping factors (i.e., all items loading approximately similarly indicating shared variance). Rather, we see several items which load lower on the general factor but load higher on the grouping factor, for example, Vocab 2, 4, 21, and 24 all have loadings above 0.5 on the grouping factor.

### Study 1 Interpretation

The first aspect to note about the estimated loadings on the unidimensional model is that they are all positive, and the majority – across both grammar and vocabulary items – are larger than 0.32, the rule of thumb value to consider loadings as statistically meaningful (Tabachnick and Fidell, 2007). For standardized solutions, this value indicates that the latent factor explains more than 10% of the variance in the item, a figure obtained by squaring the loading [see Brown (2015, p. 52) for an accessible explanation of the relationship between indicator variables and latent factors]. The fact that none of the loadings are negative indicates that all the items are measuring the latent trait in the same way, i.e., a positive response indicates higher ability. This is very much to be expected on such a rigorously designed assessment tool, and any negative loadings would be a serious cause for concern and item removal. Vocabulary items tend to load higher on the general factor than the grammar items. However, as noted above, the vocabulary items have pooled response options, which means that they will have at least some dependencies between items because of this. This may be the cause of the higher loadings, and it would be advisable to inspect correlations in the error terms further.

The correlated factor model, meanwhile, is indicated by the lower AIC and BIC to provide a more accurate description of the data. However, the high positive correlation between the two factors (*r* = 0.88) implies a poor discriminant validity between factors (Brown, 2015, p. 28), in combination with the similarities in magnitude of the loadings on the correlated two-factor model compared with the loadings in the unidimensional model mean. It is not fully clear, from a substantive perspective, what additional insight is gained from splitting the construct into two factors. Given that we would be expecting some difference purely as a result of random error, the small MAE values showing little difference in factor loading or score over and above the unidimensional model, bear out the suggestion that there seems little to be gained from reporting this particular set of items with separate grammar and vocabulary scores. While from a statistical perspective better comparative fit indices indicate a better representation of the data, when we cast it in practical terms, the improvement is minimal. It is here that the loading estimates from the bifactor model can provide additional insight.

Recall that the loadings on the skill-specific grouping factors in the bifactor model are not interpreted in the same way as those in the correlated factors model. In the bifactor model, these estimates indicate the degree of shared variance between groups of items after accounting for the general factor. In the case of the current concerns, we can discern whether there is any systematic association between grammar items, or between vocabulary items, *once the more general construct has been taken into account.* A set of strong, relatively uniform, loadings on the grouping factors would indicate a dependence between items that is not picked up on by the more general factor, i.e., something unique to vocabulary knowledge over and above the lexico-grammatical knowledge accounted for by the general factor. However, rather than consistent strong loadings for the grouping factors, the estimates show a minimal number of items with high loadings on the grouping factor, either for vocabulary or for grammar. There are four grammar items and four vocabulary items, which have a loading higher than 0.5, which indicates that 25% or more of the original observed variance is explained by the grouping factor. As a diagnostic, this indicates a deviation from the general construct among these few items. Examining the content of these two sets of items, respectively, would be recommended in real-life test construction or evaluation to identify if there are any characteristics that make them distinct from the rest of the grammar or vocabulary items. Overall, however, the mean loadings on the grouping factors are 0.21 (grammar) and 0.25 (vocabulary), which indicates that, collectively, around 5% of the observed variance in each group of items can be explained with reference to a skill-specific grouping factor, once the general lexico-grammatical ability has been taken into account.

As a researcher armed with this information, the question here is whether these discrepancies contribute something distinct enough at the point of use to merit reporting on two separate scales. Clearly, the multidimensionality route provides the statistically better fitting solution, however, is this enough to require or allow a meaningful division of the scores? There is some evidence of multidimensionality from item fit statistics. However, it was demonstrated that item fit statistics should not be the only criterion used to guide decisions, as they can be sensitive to non-construct relevant variance. It would, therefore, be acceptable to conclude that there is no compelling evidence that these items require to be reported on separate scales. Indeed, in the case of the Aptis test, the grammar and vocabulary are reported as a single score (O’Sullivan and Dunlea, 2015). This reporting structure is supported empirically in a study, which marries bifactor analysis with other methodologies to generate a battery of evidence on which to base dimensionality considerations (McCray and Dunn, in press). Treating this set of grammar and vocabulary items on the same scale can be viewed as reflecting both insights about the underlying constructs the two sets of items are designed to measure, as well as the onward consequences and application of the score. A point to note is that this decision regarding the reporting structure of this Aptis test component does not necessarily generalize all grammar and vocabulary items as operationalized in other testing scenarios.

## Illustrative Study 2 – Aptis for Teens Four Skills

In this section, we turn our attention to a commonly specified theoretical model in language testing, the four-skill model (e.g., Stricker and Rock, 2008; Sawaki et al., 2009; Sawaki and Sinharay, 2013, 2017; In’nami et al., 2016). The four-skill model posits that the receptive skills reading and listening, along with the productive skills, speaking and writing, are fundamental, divisible, and separately scorable abilities as part of the construct of general L2 ability. Here, we fit four different models: (i) unidimensional, (ii) correlated factors, (iii) higher-order, and (iv) bifactor model, and compare the inferences about underlying dimensionality, we can make from each. The models fitted are illustrated in Figure 2.

### Study 2 Dataset

The current illustrative example utilizes data from the Aptis for Teens test. This is a variant within the British Council’s Aptis suite of tests designed for the use of learners of English aged between 13 and 17 years. Further information about this test is available in the Aptis for Teens Candidate Guide (British Council, 2019b).

The scoring system for the test components is different for receptive and productive skills. Listening and reading each comprise a series of four testlets, which address a candidate’s ability to interpret an input text common to each testlet (written or aural, as relevant). Each item is scored dichotomously, though the independence assumption is violated to some extent by the testlet format. Speaking and writing, meanwhile, require the test taker to respond to a series of four tasks each, which are submitted for marking by a human rater who apportions a score to a maximum of between 4 and 7 points depending on the task. Only three tasks from the speaking component are included in the modeling exercise, as this component allocates the first task randomly from a pool of items, leading to a large degree of structural missingness. It would not be possible to retain the response data from this task for analysis without introducing inconsistencies into the analysis.

Score data analyzed in the current study is taken from a sample of 1,432 15-year-old students from the Madrid region of Spain who sat the test in 2017 as part of a wider British Council project (Shepherd and Ainsworth, 2017)^3^. Full involvement and approval of Madrid Ministry of Education was obtained prior to conducting the original study. Individual participation in the study was contingent on receiving written parental consent, with conditions agreed with the Madrid government.

### Study 2 Method

The methodology for this illustrative study follows that of study 1 (see above), with the addition that the higher-order model is also fit to this dataset. With each of the four-skill factors as first-order factors, the higher-order model is identified and, therefore, a statistically viable alternative to consider. The Mplus code used for the analysis is available in Supplementary Data Sheet 1. All four models are illustrated in Figure 2.

### Study 2 Results

Figures 2A–D give traditional factor loading diagrams for the four models used in this section. Note that they are abbreviated, in that all not all observed variables are displayed. Supplementary Table A2 in the appendix provides the full list of item loadings. For all models, the chi-square *p*-values are statically significant, as can be seen in Table 2. Again, however, this is no particular cause for concern. All models have good levels of fit on RMSEA and CFI. In terms of statistical measures of comparative fit, the best fit is achieved by the bifactor model, followed by the correlated factors, then the higher-order model, with the comparatively worst fit yielded by the unidimensional model (though, as noted, still within the acceptable thresholds on key indicators).

**TABLE 2:** Four-skills model fit and parameter comparisons.

	**Model descriptive statistics**										
**Fit measure**	**Unidimensional**	**Correlated factors**	**Bifactor**	**Higher order**						
Chisq	5627 (1430), *p* < 0.001	4255 (1424), *p* < 0.001	2454 (1375), *p* < 0.001	4273 (1426), *p* < 0.001						
RMSEA	0.045	0.037	0.023	0.037						
CFI	0.952	0.968	0.988	0.967						
AIC	89592	88384	87315	88446						
BIC	90329	89152	88612	89204						
ADJ BIC	89884	88687	87992	88746						

**Mean (SD) of factor loadings on observed variables**									**Loadings on higher-order factor**	

General	0.62 (0.15)	*NA*^a^	0.62 (0.15)	0.93 (0.42)					*NA*^b^	
Listening	*NA*^a^	0.58 (0.19)	0.13 (0.11)	0.58 (0.19)					0.97	
Reading	*NA*^a^	0.67 (0.14)	0.13 (0.27)	0.67 (0.14)					0.90	
Speaking	*NA*^a^	0.89 (0.02)	0.45 (0.04)	0.89 (0.02)					0.89	
Writing	*NA*^a^	0.74 (0.10)	0.27 (0.08)	0.75 (0.10)					0.96	
**Factor Correlations**					
LI-RE	*NA*^c^	0.90 (0.01)	*NA*^c^	*NA*^c^						
LI-SP	*NA*^c^	0.86 (0.01)	*NA*^c^	*NA*^c^						
LI-WR	*NA*^c^	0.91 (0.01)	*NA*^c^	*NA*^c^						
RE-SP	*NA*^c^	0.76 (0.01)	*NA*^c^	*NA*^c^						
RE-WR	*NA*^c^	0.86 (0.01)	*NA*^c^	*NA*^c^						
SP-WR	*NA*^c^	0.89 (0.01)	*NA*^c^	*NA*^c^						
**Mean Absolute Error (MAE)^d^ of parameterscompared toequivalent(s) in unidimensional model**										
	**Correlated factors**				**Bifactor**	**Higher order**				
	**Li**	**Re**	**Sp**	**Wr**	**Gen**	**Gen**	**Li**	**Re**	**Sp**	**Wr**

Factor loadings	0.02	0.03	0.09	0.03	0.02	*NA*^f^	0.01	0.03	0.09	0.04
Factor scores^e^	0.9	1.7	7.2	3.9	1.24	1.02	*NA*^g^	*NA*^g^	*NA*^g^	*NA*^g^

*^*a*^Not provided as the factor was not measured by the model. ^*b*^No loading of general factor on itself. ^*c*^Not provided as no correlation between factors measured in the model. ^*d*^$M\,A\,E=\frac{\sum \left|x_{i}-y_{i}\right|}{n}$ where x is a parameter for the unidimensional model, y is a corresponding parameter from the model in question, and n is the sum of comparisons. ^*e*^Scores set to mean = 50, SD = 10. ^*f*^Not provided as items do not load directly onto the general factor. ^*g*^Not provided as the results of the higher order factor are analogous to those on the unidimensional model, not those of the lower order factors.*

The average loading of items on the unidimensional model is 0.62, providing a mean explanation of 38% of the variance of the observed variables. This relatively high loading indicates that meaningful measurement of the construct is taking place (Tabachnick and Fidell, 2007). For the correlated factors model, the average loading of the speaking (0.89) and writing (0.74) are higher than that of the reading (0.67) and listening (0.58) items. This is likely a consequence of the polytomous nature of the response options for the productive skills, generating fewer but strong correlations with the general factor rather than the many weaker, yet still informative, correlations seen in the binary items used in the receptive skills. The average loadings for the subordinate factors in the higher-order model are virtually the same as those in the correlated factors model, unsurprisingly. All four first-order factors load very highly (0.89–0.97) on the general L2 ability factor. In the bifactor model, the average loading on the general factor is very close to that of the unidimensional model (0.62), however, the mean loadings for the grouping factors from 0.27 (listening) to 0.45 (speaking) indicate that there may be persuasive evidence of multidimensionality for some of the grouping factors. In terms of loading MAE scores, there is a large difference between the loadings on the unidimensional model and those on the speaking factor (0.09) of the correlated factors and second-order models. Regarding the MAE values for score, there is a sizeable difference between the scores of the unidimensional model versus those on the speaking (7.2) and, to a lesser extent, writing (3.9) components of the correlated factors model.

### Study 2 Interpretation

As mentioned above, the fits statistics indicate that all the models presented offer a reasonable explanation of the data (CFI > 0.95; RMSEA < 0.06). In effect, any confirmatory question we ask about the dimensionality of the test as modeled by any of these models we could justify statistically. In this situation, the value of the different models lies in the information they give us about the comparative ways of handling the dimensionality of the test. For example, based on model fit statistics alone, if we were to ask the question “Is this test unidimensional enough to treat on a single scale?” We would cite RMSEA 0.045 and CFI 0.952 and answer “yes.” However, as we saw above, selecting purely based on comparative fit statistics is likely unwise. The difference between speaking and writing compared with general L2 ability scores from the unidimensional model highlighted by the MAE value indicates a practical need to report scores on more than one dimension. The general L2 ability score is not a suitable proxy for the writing and, to a lesser extent, speaking skills measured by Aptis.

The latent variables in the correlated factors model all correlate strongly, the highest being between writing and listening (*r* = 0.91), and the lowest between speaking and reading (*r* = 0.76). The comparative fit of the higher-order model is not favorable to the correlated factors model, with a marginally lower score on each of the information criterion. Given the good global fit statistics, however (RMSEA 0.037; CFI 0.967), again, in a confirmatory factor analytic approach, this higher-order model would be accepted as a solid way of understanding the factor structure. It is clear from the strong positive loadings of the higher-order factor on the four subordinate factors that this factor is a good summary of the four skill areas, with very little associated error.

At this point, if the researcher is interested in investigating the nature of the multidimensionality further, he or she may wish to model the data using a bifactor model. Looking to the loadings for the skill-specific grouping factors in the bifactor model for these data, a number of points can be observed. The first is that all three speaking tasks have loadings of greater than 0.32 on the grouping factor, with a mean of 0.45. This shows that the grouping factor is explaining more than 20% of the observed variance across these task responses, which is suggestive of a systematic deviation from the variance explained by the general factor. While the mean loadings on the grouping factor for writing are not as high at 0.27, it is still markedly higher than the mean loadings for reading and listening at 0.13. In some respects, it is not unexpected to see this pattern, given the role of individual difference in explaining performance in tests of the productive skill areas (see, e.g., Kim and Crossley, 2019). The other grouping factor with several items loading higher than 0.32 is reading. In this case, however, this pattern is only observed for items associated with “task 2” in the reading component. This indicates that individual item responses associated with this particular task have a strong dependency distinct from the explanation provided by the general factor^4^. In this respect, the bifactor model has highlighted a source of systematic construct irrelevant variance.

This brief illustration has shown the dual usefulness of the bifactor model in estimating the magnitude of dependencies between sub-groups of items beyond the general ability hypothesized. The additional variance on the speaking and writing tasks may validly be attributable to a feature of test performance that is distinct from the overall L2 ability represented by the general factor. Meanwhile, in the case of the reading test, we see an example of what Reise et al. (2010) highlight as *nuisance* dimensions – “factors arising because of content parcels that potentially interfere with the measurement of the main target construct” (Reise et al., 2010, p. 5). In cases where the skill-specific grouping factors indicate substantial degree of shared variance over and above the explanation provided by the general factor, it rests on the researcher to bring their knowledge of the item content and the context of testing to the interpretation, and ultimately whether this is viewed to be worth accounting for separately.

## Discussion

The two illustrative examples presented above show how dimensionality might be assessed in language testing-specific contexts using CFA models. In the first example, looking at grammar and vocabulary items, evidence of multidimensionality was indicated by model fit statistics. However, it was shown in various ways, e.g., similar loading and factor scores between uni- and multidimensional models, that the practical ramifications of ignoring that multidimensionality would be small. Furthermore, the dominance of a small number of items on the bifactor skill-specific grouping traits led to the conclusion that there would be little to be gained from splitting and reporting separate scores for grammar and vocabulary in this case. The second illustrative study examined the dimensionality of the four-skill model. Again, evidence from item fit indicated multidimensionality. However, in this example, the need to report sub-scores for the productive skills (i.e., speaking and writing) was indicated by the fit of the correlated factor model, the high loadings on some bifactor grouping traits, and the large differences between the MAE values on factor loading and score for some subscales. In both cases, the bifactor model, alongside the more traditionally fitted models greatly aided the evidence-gathering process. This highlights a central methodological point, that rather than viewing the bifactor model as providing an opposing latent structure to test against, it should be understood as providing the researcher with the capacity to investigate the assumption of a combination of general and skill-specific abilities more thoroughly. This is consistent with the first of our standpoints stated at the outset of the studies, encouraging a move away from the approach, which asks, “which CFA model fits the data best?” toward understanding each CFA model as a tool, offering related, but distinct, insights to the researcher. In fact, the nesting relationship between the bifactor model and higher-order model already alluded to in this paper, shows the bifactor to be the less restricted of the two models. The bifactor model is, therefore, often able to more flexibly account for variance in the data than the higher-order model, and is thus more likely to yield favorable fit statistics when modeling real-world data with potentially unaccounted for complexities (for a more detailed explanation, see Yang et al., 2017).

Recalling the second standpoint posited at the beginning of the paper, it has been advanced here that the researcher is best placed to initiate their investigation from an understanding that tests are not either unidimensional *or* multidimensional, but that all tests with more than one item are multidimensional to some extent (Gustafsson and Åberg-Bengtsson, 2010; Reise et al., 2010). Echoing Reise et al.’s statement that, “when a scale is subjected to “confirmatory” factor analyses, the conclusion is, almost without exception, that the data are multidimensional” (Reise et al., 2010, p. 16), we found evidence of multidimensionality from the comparison of fit statistics between the unidimensional and multidimensional models in both illustrative studies. The four models presented and discussed in this paper, rather than being viewed as competitors in providing the *best* explanation of a dataset, via model selection of minimal AIC/BIC or some other criterion, can be seen as tools to be employed in exploring and understanding the latent structure of a test. We would suggest, again in line with Reise et al. (2010), that some method of assessing the practical impact of multidimensionality be undertaken. In practice, this means answering dimensionality questions by scrutinizing the nature and relative size of loading estimates rather than solely through comparisons of model fit. As illustrated in the studies described above, this could take the form of looking at the differences in loadings in scores between the uni- and correlated factors model, or, equally, by examining the size and distribution of the loadings on the grouping factors of the bifactor model. Relatively uniform loadings on the grouping factor indicate score variance common to all subscale items that is untapped by the general factor. The magnitude of the loadings is reflective of the extent to which reporting a separate score for that factor is important. Non-uniform loadings indicate correlations between specific items, which should be investigated further. This level of detail enables the researcher to pick up on nuances that are not so easily discernible from higher-order model estimates^5^.

To elaborate further using the example of modeling four-skill data, employing the higher-order model in a CFA framework has often been a natural step to take, since this factor structure provides an intuitive reflection of the current theoretical conceptions of language tests (Stricker and Rock, 2008; Sawaki et al., 2009; Harsch, 2014). In order to understand the closeness of the relationship between the sub-skills and the overarching factor in this model, the researcher will look to the disturbance estimates (the error associated with the first-order factors) against each of the subordinate factors. In fact, these disturbance estimates in the higher-order factor model are analogous to the skill-specific grouping factors in the bifactor model (Reise et al., 2010, p. 5). From the single disturbance estimate for each skill area, we would have been able to discern slightly larger overall disturbance estimates for writing and, in particular, speaking, compared to the other skill areas. This could lead to the same conclusion regarding a distinct underpinning of the speaking, and also writing components, perhaps due to individual differences. However, from a single disturbance estimate, it would not be possible to identify clusters of items within an individual skill area that might be the driving source of additional variance. This was seen in the four-skill bifactor model above, where reading items grouped in a single testlet displayed an interdependence distinct from the general factor. This extra information about individual observed variables highlights the added value from the bifactor model.

Broadening this out to a consideration of how the bifactor model can be understood as complimentary to the higher-order model, it is useful to bring in considerations of the similarities between the two models. As observed by Markon, “These two paradigms differ in how levels of abstraction are modeled: In one, superordinate factors are at a greater level of abstraction because they influence subordinate factors; in the other, superordinate factors are at a greater level of abstraction because they influence a greater breadth of observed variables” (Markon, 2019, p. 53). This thinking is also presented by Gustafsson and Åberg-Bengtsson (2010). These researchers suggested that it is a misconception to distinguish between the two models based on the differing “distance from reality” of the general factors, i.e., whether they load directly on the observed variables. They highlight the fact that both models share two types of factors, exerting broad and narrow influence, respectively, with the key difference between models lying on whether a simple or complex structure is retained, rather than any fundamental distinction in theoretical underpinnings. While on the face of it, the bifactor model is more complex as a latent structure than the higher-order model, the interpretation of the variance explanation becomes much more straightforward, owing to the clear separation between general and grouping factors. The bifactor model can, therefore, be recognized as a powerful means of assessing multidimensionality assumptions, in a manner that is consistent with current theoretical understandings of the latent structure of language tests.

When making a decision about how scores are to be reported, it should equally be recognized that the statistical evidence is only one consideration. Researchers should look to both explore the observed properties of the responses, as well as to establish a structure that suits the data in the light of the uses and interpretations that the test score report will need to fulfill. Often, the expectation of score users, whether rightly or wrongly, may trump the measurement considerations. For example, although we found evidence in illustrative study 2 that there is no prohibition against reporting the receptive skills on the same scale, doing so would represent such a break with conventions that is unlikely to be implemented in a large-scale test in practice. We agree with Rijmen (2010) who comments that good statistical practice should balance the modeling and empirical fit considerations with substantive theory. However, we would argue that in language testing, stakeholder expectations also need serious consideration.

## Conclusion

This paper illustrated how the bifactor model can be used alongside other traditionally employed psychometric models to assess the underlying dimensional structures of the construct(s) measured by a test. Fundamentally, the bifactor model lets the researcher look in detail at what variance is common in a subscale that is *not* explained by a general factor. An examination of the patterns and magnitudes of the loadings not explained by a general factor is tremendously valuable for assessing the weight of evidence for uni- or multidimensionality and also for diagnosing problematic groups of items. Through the illustrative examples, each of which came to substantively different conclusions about the dimensionality of the test, it is hoped that a template for the usage of the bifactor model in language testing research has been provided, and recommendations have been given on how to approach inference from the model. We have argued for, and hope to see, a more multifaceted approach to dimensionality assessment through CFA in the future that not only takes account statistical model fit and theoretical pre-suppositions but also considers the practical impact of score/sub-score reporting and stakeholder expectations of what will be reported in the final analysis.

## Data Availability Statement

Commercial confidentiality means the data are not publicly available. However, access to Aptis test data is available for research purposes to British Council Assessment Research Grant holders. Further information at: https://www.britishcouncil.org/exam/aptis/research/assessment-advisory-board/awards/assessent-grants.

## Author Contributions

KD and GM made substantial contributions to the conception of the project, drafting and critical redrafting of the manuscript. KD ran the statistical models. Both authors worked in detail on individual aspects of the analysis and interpretation.

## Conflict of Interest

The authors declare that the research was conducted in the absence of any commercial or financial relationships that could be construed as a potential conflict of interest.

## Footnotes

1 Note that in the bifactor model, it is not the case that a unidimensional model is fitted, and then group factors are fitted to the residuals. Rather, the general and specific factors are fitted at the same time, and thus, the specific factors are only analogous to residuals.

2 $M\,A\,E=\frac{\sum \left|x_{i}-y_{i}\right|}{n}$, where x is a parameter for the unidimensional model, y is a corresponding parameter from the model in question, and n is the sum of comparisons.

3 Not all cases from the original study were available for item-level analysis, so this represents a sub-set of the full project dataset. Exclusions were related to a technical aspect of version allocation and were not contingent on candidate-related factors.

4 The scoring approach taken for this particular testlet was, in fact, revised since the recording of the scores in the current dataset, owing to issues with dependence and the representation of construct (Spiby and Dunn, 2018).

5 Unless transformed using the Schmid–Leiman transformation referenced above. Regardless, the higher-order model imposes a greater degree of constraint on the relationship between the general factor and the observed variables (Markon, 2019).
