# Supplementary material for: The Place of the Bifactor Model in Confirmatory Factor Analysis Investigations Into Construct Dimensionality in Language Testing
Source: Front Psychol. 2020 Jul 17;11:1357. doi: 10.3389/fpsyg.2020.01357 (PMC7379375; doi:10.3389/fpsyg.2020.01357)
Supplement: Supplementary file 1 [file Data_Sheet_1.docx]

**Mplus code**

**TITLE:** Study 2 Analysis;

! REMOVE EXCLAMATION MARKS TO ACTIVATE RELEVANT LINES OF CODE

**DATA:** FILE IS Data.txt;

**VARIABLE:** NAMES ARE

Cand_ref L_01 L_25 R_01_1 R_04_7 S_02 S_04 W_01 W_04; !TRUNCATED VAR LIST

USEVARIABLES ARE

L_01 - W_04;

CATEGORICAL ARE

L_01 - W_04;

MISSING ARE ALL (9999);

IDVARIABLE = Cand_ref;

**MODEL:**

! !!BIFACTOR MODEL

GEN BY L_01* - W_04;

SL BY L_01* - L_25;

SR BY R_01_1* - R_04_7;

SS BY S_02* - S_04;

SW BY W_01* W_04;

G@1;

SL@1;

SR@1;

SS@1;

SW@1;

G WITH SL@0 SR@0 SS@0 SW@0;

SL WITH SR@0 SS@0 SW@0;

SR WITH SS@0 SW@0;

SS WITH SW@0;

! !!CORRELATED FACTORS MODEL

! LI BY L_01 - L_25;

! RE BY R_01_1 - R_04_7;

! SP BY S_02 - S_04;

! WR BY W_01* W_04;

! !!HIGHER-ORDER MODEL

! LI BY L_01 - L_25;

! RE BY R_01_1 - R_04_7;

! SP BY S_02 - S_04;

! WR BY W_01* W_04;

! GEN BY LI RE SP WR;

! !!UNIDIMENSIONAL MODEL

! G BY L_01 – w04;

**ANALYSIS:**

! !!WLSMV WITH THETA PARAMETERISATION

parameterization=theta;

estimator=wlsmv;

! !!MLR PROBIT LINK MODEL

! LINK=PROBIT;

! ESTIMATOR=MLR;

**OUTPUT:**

STANDARDIZED;

TECH4;

**SAVEDATA:**

FILE is FILENAME.txt;

SAVE = FSCORES;
